# Profiling and functional analysis of differentially expressed circular RNAs in high glucose‐induced human umbilical vein endothelial cells

**DOI:** 10.1002/2211-5463.12709

**Published:** 2019-08-22

**Authors:** Guoxi Jin, Qiong Wang, Xiaolei Hu, Xiaoli Li, Xiaoyan Pei, Erqin Xu, Minglong Li

**Affiliations:** ^1^ Department of Endocrinology First Affiliated Hospital of Bengbu Medical College Anhui China; ^2^ Room of Physical Diagnostics Bengbu Medical College Anhui China; ^3^ Department of Endocrinology Shandong Provincial Hospital Affiliated to Shandong University Jinan Shandong China

**Keywords:** circRNA–miRNA network, circular RNA, diabetes, diabetic vascular damage, endothelial dysfunction, human umbilical vein endothelial cells

## Abstract

Dysfunction of vascular endothelial cells often results in diabetic vascular complications. Circular RNAs (circRNAs) have been implicated in the pathogenesis of various diseases, including diabetes and many vascular diseases. This study aimed to explore the roles of circRNAs in high glucose‐induced human umbilical vein endothelial cells (HUVECs) to elucidate the contributions of circRNAs to diabetic vascular complications. We subjected control and high glucose‐induced HUVECs to RNA sequencing and identified 214 differentially expressed circRNAs (versus control HUVECs, fold change ≥ 2.0, *P* < 0.05). We then validated seven of these differentially expressed circRNAs by qPCR (*hsa_circ_0008360*, *hsa_circ_0005741*, *hsa_circ_0003250*, *hsa_circ_0045462*, *hsa_circ_0064772*, *hsa_circ_0007976*, and *hsa_circ_0005263*). A representative circRNA–microRNA (miRNA) network was constructed using the three most up‐regulated circRNAs (*hsa_circ_0008360*, *hsa_circ_0000109*, and *hsa_circ_0002317*) and their putative miRNA. Bioinformatic analysis indicated that these circRNAs regulate the expressions of genes involved in vascular endothelial function and angiogenesis through targeting miRNAs. Our work highlights the potential regulatory mechanisms of three crucial circRNAs in diabetes‐associated endothelial dysfunction.

AbbreviationsCircRNAs: circular ribonucleic acid
GO: Gene Ontology
HUVECs: human umbilical vein endothelial cells
KEGG: Kyoto Encyclopedia of Genes and Genomes
MF: molecular function
miRNA: microRNA
MREs: miRNA response elements
NF‐kB: nuclear factor kappa B
QC: quality control
T2DM: type 2 diabetes mellitus
VEGF: vascular endothelial growth factor

Type 2 diabetes mellitus (T2DM) is a chronic metabolic disease featured with high blood glucose level. Patients with T2DM often suffer from a variety of complications associated with vascular disease, including vascular smooth muscle cell dysfunction, atherosclerosis, cardiomyopathy, and nephropathy 1, 2, 3. Dysfunction of endothelial cell and their imbalanced proliferation and apoptosis are the primary pathological mechanisms of diabetic vascular complications 4, 5. Extensive studies have revealed that various factors, including aging, obesity, hypertension, hyperlipidemia, hyperglycemia, low‐grade inflammation, and insulin resistance, play a significant role in endothelial dysfunction in T2DM 6, 7, 8. Since vascular endothelial cells play a major role in maintaining cardiovascular homeostasis, more investigations are now focused on effective approaches to prevent hyperglycemia‐induced endothelial cell injury 9, 10, 11.

Circular RNAs (circRNAs), a class of endogenous, noncoding RNAs that form covalently closed continuous loops 12, 13, 14, are known to be predominantly produced by backsplicing reactions that covalently link the 3′ end of an exon to the 5′ end of an upstream exon 15, 16, 17. Widely expressed in eukaryotes, circRNAs can be derived from exon, intron, untranslated, or intergenic regions of the genome, and a large amount of circRNAs have been identified in mouse and human by high‐throughput sequencing and bioinformatics analyses 18. Among multiple functions of circRNAs unveiled till now, the most important one is the involvement of circRNAs in gene post‐transcriptional regulation. As well known, circRNAs can influence gene expression in mammals as miRNA sponges through interacting with the miRNA response elements (MREs) 19. Since circRNAs have been found to be widely involved in atherosclerotic vascular disease 20 and diabetes 21, 22, it is interesting to explore the effect of high glucose‐induced dysfunction of circRNAs on vascular endothelial cells. Human umbilical vein endothelial cells (HUVECs) are cells derived from the endothelium of umbilical cord veins and are considered as a good model system for studying the molecular mechanisms in diabetes and atherosclerosis 23. However, up to now, there are few studies that have systematically investigated the alterations of global circRNA expression in high glucose‐induced HUVECs.

In this study, to explore the implications of circRNAs in high glucose‐induced endothelial dysfunction, we conducted RNA sequencing (RNA‐seq) in control HUVECs and HUVECs induced with a high concentration of glucose. A total of 214 circRNAs were identified to be differentially expressed in high glucose‐induced HUVECs, and quantitative PCR assays verified the altered expression of selected circRNAs. Moreover, comprehensive bioinformatics analyses were performed to speculate and probe the functions of the parental genes of these differentially expressed circRNAs. Furthermore, our analyses on the circRNA/microRNA (miRNA) interaction network underscored the potential functional profiles of high glucose stimulation‐associated circRNAs in human vascular endothelium.

## Materials and methods

### Cell culture and RNA sequencing sample preparation

Culturing of HUVECs and RNA preparation were broadly followed in the previous study 24. HUVECs were obtained from China Center for Type Culture Collection (CCTCC, Wuhan, China) and were maintained in modified Eagle's medium (MEM; HyClone/Thermo Fisher Scientific, Waltham, MA, USA) supplemented with 10% FBS (Gibco, Gaithersburg, MD, USA), 100 units·mL of penicillin, and 100 µg·mL^−1^ of streptomycin (Gibco), and grown in a humidified atmosphere with 5% CO_2_ at 37 °C. High glucose‐induced HUVECs were cultured in medium containing 25 mm glucose for 6 days, and control HUVECs were cultured with 5 mm glucose and 20 mm mannitol for the same duration.

Total RNA was isolated and purified using the TRIzol reagent (Invitrogen, Carlsbad, CA, USA) following the manufacturer’s instructions. The quantity and quality of the RNA samples were determined using NanoDrop 2000 (Thermo Fisher Scientific) and Agilent 2100 Bioanalyzer (Agilent Technologies, Santa Clara, CA, USA).

### High‐throughput RNA sequencing of circRNA

TruSeq® Stranded Total RNA Sample Preparation Kit was used for cDNA library preparation in accordance with the manufacturer’s instructions. First, Ribo‐Zero rRNA removal beads were used to remove ribosomal RNA. Next, the RNA was purified and fragmented into small pieces. The double‐stranded cDNA fragments were syntheses which were copied from cleaved RNA fragments. These cDNA fragments then underwent a series of approaches including the end repair process, the addition of a single ‘A’ base, and ligation of the adapters. The products were purified and enriched with PCR to generate the final cDNA libraries. Purified libraries were quantified by Qubit® 2.0 Fluorometer (Thermo Fisher Scientific) and Agilent 2100 Bioanalyzer (Agilent Technologies). Cluster was generated by cBot with the library diluted to 10 pm and sequenced by the Illumina HiSeq 2500 (Illumina, San Diego, CA, USA). The sequencing was performed at Origin Biotech Inc. (Ao‐Ji Biotech, Shanghai, China).

### Bioinformatics analysis

Quality control (QC) of RNA‐seq reads was conducted with FastQC (v. 0.11.3). Reads were trimmed using the software seqtk for known Illumina TruSeq adapter sequences, poor reads, and ribosome RNA reads. The trimmed reads were then mapped to the Homo sapiens reference genome (hg38) by the BWA‐MEM (version 2.0.4). The circRNAs were predicted via CIRI software 25, and circRNAs that were perfectly matched to circBase were considered as known circRNAs 26. Normalization of counts was performed by SRPBM 27. Differentially expressed circRNAs were analyzed using edgeR 28. The circRNAs satisfied with fold changes ≥ 2, *P*‐values < 0.05 were considered differentially expressed. In order to identify the association between circRNAs and their related miRNAs, the significantly altered circRNAs between high glucose‐induced and control HUVECs were used to predict the circRNA–miRNA interactions using the Ao‐Ji Biotech’s homemade software that was based on miRanda 29, and circRNA–miRNA network was constructed by Cytoscape 30. Top 10 significantly up‐regulated and down‐regulated circRNAs were listed with MREs.

### Real‐time quantitative reverse transcription–polymerase chain reaction (qRT‐PCR)

Total RNA was extracted and reversely transcribed into cDNA using SuperScript™ III Reverse Transcriptase (Thermo Fisher Scientific). The expression levels of seven randomly selected circRNAs that differentially expressed in high glucose‐induced HUVECs were determined by FastStart Universal SYBR Green Master (Rox) with specific primers (Table 1). PCRs were performed in triplicate with the following temperature profile: denaturation at 95 °C for 10 min, followed by amplification for 40 cycles with each cycle consisting of 95 °C for 10 s and 60 °C for 1 min. *GAPDH* was used as an internal reference gene. Data were analyzed using $2^{-\Delta \Delta C_{t}}$ method, and the expression level of each circRNA was represented as the fold change between the control group and high glucose‐induced group.

**Table 1:** The sequences of PCR primers used in this study.

Gene name	Primers sequences (5′–3′)	PCR product length (bp)
*GAPDH*	CCTGGTATGACAACGAATTTG	131
	CAGTGAGGGTCTCTCTCTTCC	
*hsa_circ_0008360*	TGGAGTAGACGAAGCCTATACG	158
	CTGTCCCACTCTGCCCTTG	
*hsa_circ_0005741*	GCTCTTTTGTGACAGGGACG	151
	TTCAGCAGAATCCCCTCTCG	
*hsa_circ_0003250*	GGTGAATATGGCCAGCTTCC	150
	TCACTCTGGTTTGGGACTGT	
*hsa_circ_0045462*	ACAAGTAATCACAGGGCCTCA	150
	TTGCAATCACTGTCGGCTTC	
*hsa_circ_0064772*	CCGGGTGAGAGCAAAACTTT	157
	TGCAGCTGATGATGGCCTAT	
*hsa_circ_0007976*	TGAGAGAAATGCTTACACACAGA	231
	ACAAAACCATCCAAGGCTTTCA	
*hsa_circ_0005263*	AGCCACCAGAACCTTAAGCT	167
	GGGCTTGCACTGATCTGGT	

### Statistics

Results from experiments with replicates are expressed as the mean ± SEM (standard error of the mean), and data analysis was performed by Statistical Program for the Social Sciences (spss) 22.0 software (SPSS, Chicago, IL, USA). For analysis involving only two groups, data were analyzed with Student’s *t*‐test. A *P* value less than 0.05 was regarded as statistically significant.

## Results

### RNA‐seq identified differentially expressed circRNAs in high glucose‐induced HUVECs

**Figure 1:**
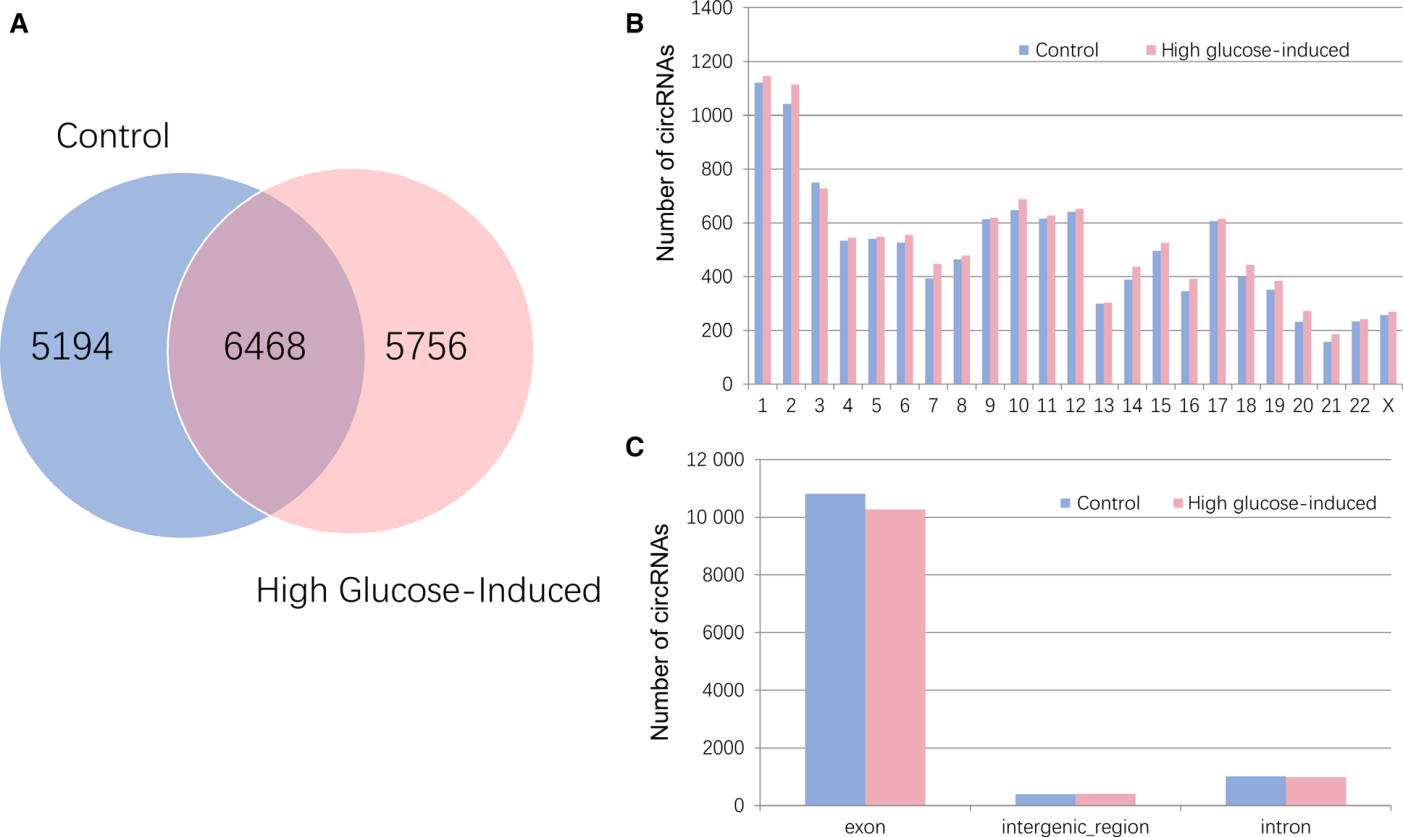
Overview of circRNAs identified in control HUVECs and high glucose‐induced HUVECs. (A–C) HUVECs cultured in medium with normal glucose concentration (5 mm, control) or high glucose concentration (25 mm, high glucose‐induced) for 6 days were subjected to RNA‐seq in order to profile the expression of circRNAs. (A) The Venn diagram depicts the numbers of circRNAs identified in control and high glucose‐induced HUVECs groups. (B) The bar graph shows the distribution of all identified circRNAs in human chromosomes. (C) The bar graph shows the source category of all identified circRNAs. Most of the circRNAs originate from the protein‐coding exons. A small portion of circRNAs is from introns, while a very few are from the intergenic region sources.

In order to characterize the landscape of circRNA expression, we performed deep RNA‐seq experiments using three normal HUVECs samples and three high glucose‐induced HUVECs samples. In total, we identified 17 418 circRNAs from six samples, and 6468 circRNAs were detected in both control and high glucose‐induced groups (Fig. 1A). These identified circRNAs are distributed on all the human chromosomes, and chromosomes 1 and 2 have more circRNAs than other chromosomes (Fig. 1B). Furthermore, we analyzed the category of these circRNAs. The vast majority of them are transcribed from the protein‐coding exons, while the others are transcribed from introns and intergenic regions (Fig. 1C).

**Figure 2:**
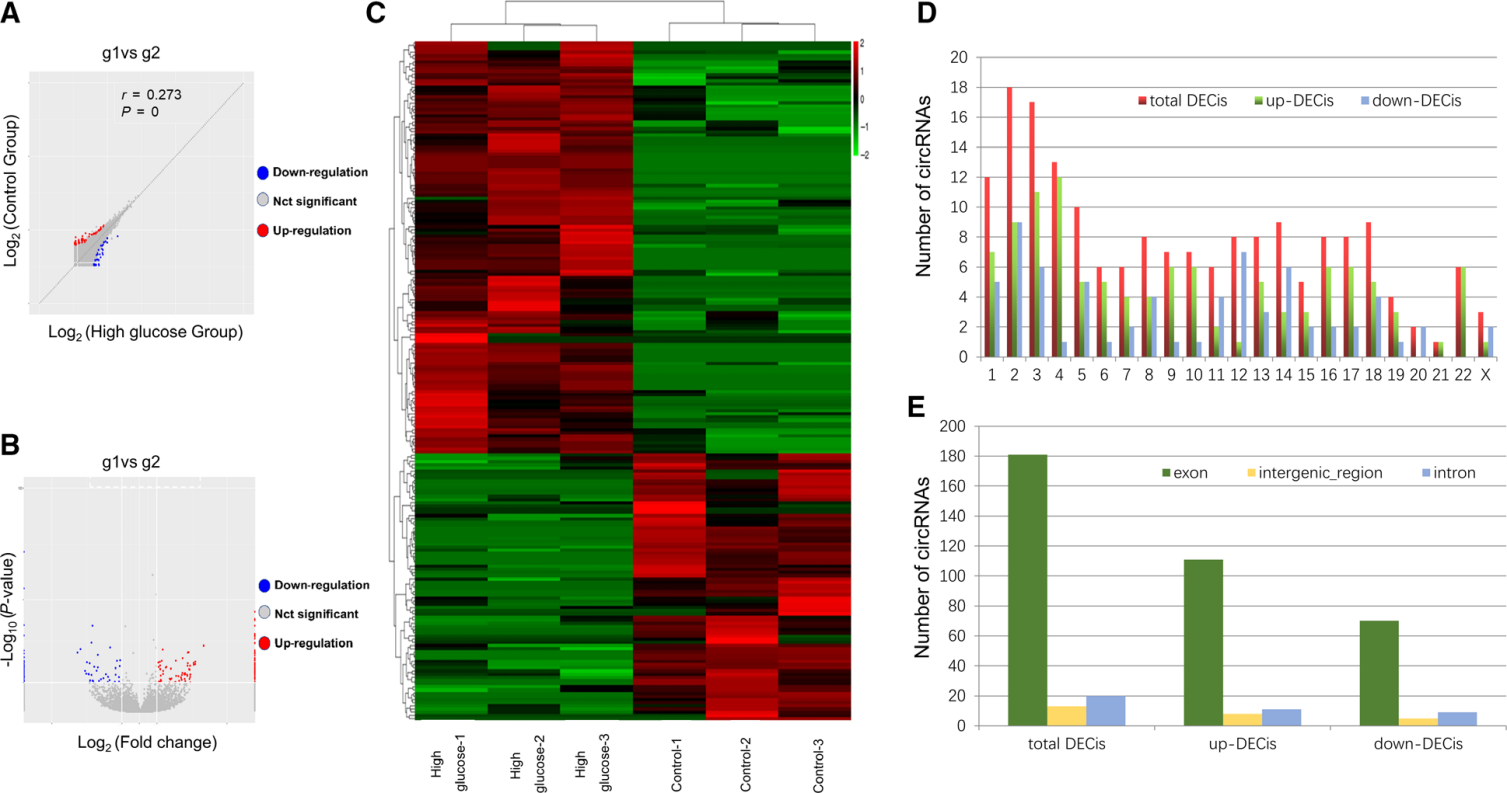
General characterizations of differentially expressed circRNAs in high glucose‐induced HUVECs. (A, B) Differentially expressed circRNAs were displayed by the scatter plot (A) and volcano plot (B). The blue and red dots indicate the dysregulated circRNAs with > 2‐fold decreased and increased expression in high glucose‐induced HUVECs, respectively (*P* < 0.05). The gray dots indicate the circRNAs with no significant alterations in expression levels. (C) Hierarchical clustering of differentially expressed circRNAs in the samples used for the RNA‐seq assay. (D) The bar graph shows the distribution of differentially expressed circRNAs in human chromosomes. (E) The bar graph shows the source category of differentially expressed circRNAs.

The edgeR analysis of the RNA‐seq data distinguished a total of 214 circRNAs differentially expressed in high glucose‐induced HUVECs (versus control HUVECs, |fold change| ≥ 2.0, *P* < 0.05), among which 130 circRNAs were up‐regulated, whereas 84 were down‐regulated. As illustrated in Fig. 2, the scatter plot (Fig. 2A), volcano plot (Fig. 2B), and hierarchical clustering (Fig. 2C) revealed that the expression profiles of circRNAs between control HUVECs and high glucose‐induced HUVECs were diverse. The top 10 up‐ and down‐regulated circRNAs are listed in Table 2. The differentially expressed circRNAs are from all human chromosomes (Fig. 2D), and most of them are transcribed from protein‐coding exons (Fig. 2E).

**Table 2:** The top 10 up‐regulated and down‐regulated differentially expressed known circRNAs in high glucose‐induced HUVECs. FC, fold changes compared with control HUVECs; MRE, miRNA response element.

CircRNA ID	Known circRNA	Locus	CircRNA type	*P*‐value	FC	Up/Down	Gene symbol	No. of miRNA targets	MRE1	MRE2	MRE3	MRE4	MRE5
CircRNA.15166	*hsa_circ_0064772*	Chr3	Exon	0.01481	8.59	Down	CLASP2	53	*hsa‐miR‐6804‐3p*	*hsa‐miR‐424‐5p*	*hsa‐miR‐370‐3p*	*hsa‐miR‐5586‐5p*	*hsa‐miR‐93‐3p*
CircRNA.7966	*hsa_circ_0047698*	Chr18	Exon	0.01986	8.1835	Down	ME2	9	*hsa‐miR‐150‐3p*	*hsa‐miR‐1301‐3p*	*hsa‐miR‐330‐5p*	*hsa‐miR‐2278*	*hsa‐miR‐6781‐3p*
CircRNA.3075	*hsa_circ_0000398*	Chr12	Exon	0.00619	7.9646	Down	SLC38A1	9	*hsa‐miR‐513a‐3p*	*hsa‐miR‐514a‐3p*	*hsa‐miR‐221‐5p*	*hsa‐miR‐6079*	*hsa‐miR‐33b‐3p*
CircRNA.12564	*hsa_circ_0056891*	Chr2	Exon	0.02137	7.3857	Down	COBLL1	101	*hsa‐miR‐6779‐5p*	*hsa‐miR‐612*	*hsa‐miR‐6764‐5p*	*hsa‐miR‐4663*	*hsa‐miR‐1‐3p*
CircRNA.4470	*hsa_circ_0007976*	Chr14	Exon	0.0499	7.1483	Down	HIF1A	5	*hsa‐miR‐4677‐3p*	*hsa‐miR‐4802‐3p*	*hsa‐miR‐4768‐5p*	*hsa‐miR‐6833‐3p*	*hsa‐miR‐892a*
CircRNA.20816	*hsa_circ_0087015*	Chr9	Exon	0.00083	6.6675	Down	RNF38	119	*hsa‐miR‐6894‐5p*	*hsa‐miR‐4651*	*hsa‐miR‐608*	*hsa‐miR‐6756‐5p*	*hsa‐miR‐920*
CircRNA.19392	*hsa_circ_0002663*	Chr8	Exon	0.02257	6.6523	Down	C8orf76	27	*hsa‐miR‐526b‐5p*	*hsa‐miR‐3135b*	*hsa‐miR‐6877‐3p*	*hsa‐miR‐4727‐5p*	*hsa‐miR‐615‐3p*
CircRNA.3836	*hsa_circ_0005263*	Chr13	Exon	0.00014	6.3985	Down	FNDC3A	32	*hsa‐miR‐6796‐5p*	*hsa‐miR‐5006‐5p*	*hsa‐miR‐4524b‐3p*	*hsa‐miR‐3690*	*hsa‐miR‐4457*
CircRNA.2838	*hsa_circ_0025638*	Chr12	Exon	0.01456	5.4303	Down	RASSF8	37	*hsa‐miR‐1266‐3p*	*hsa‐miR‐3925‐3p*	*hsa‐miR‐4522*	*hsa‐miR‐4724‐3p*	*hsa‐miR‐3922‐3p*
CircRNA.3121	*hsa_circ_0003664*	Chr12	Exon	0.01062	5.1784	Down	LARP4	11	*hsa‐miR‐29b‐2‐5p*	*hsa‐miR‐7161‐3p*	*hsa‐miR‐7848‐3p*	*hsa‐miR‐1255a*	*hsa‐miR‐608*
CircRNA.12079	*hsa_circ_0008360*	Chr22	Exon	0.00112	12.7096	Up	XPNPEP3	21	*hsa‐miR‐7107‐3p*	*hsa‐miR‐6753‐3p*	*hsa‐miR‐18a‐3p*	*hsa‐miR‐6754‐5p*	*hsa‐miR‐186‐3p*
CircRNA.9089	*hsa_circ_0000109*	Chr1	Exon	0.00583	9.1541	Up	CAPZA1	16	*hsa‐miR‐370‐5p*	*hsa‐miR‐4731‐5p*	*hsa‐miR‐5189‐3p*	*hsa‐miR‐6760‐3p*	*hsa‐miR‐3620‐5p*
CircRNA.5846	*hsa_circ_0002317*	Chr16	Intergenic region	0.00754	9.1384	Up	n/a	497	*hsa‐miR‐4728‐5p*	hsa‐miR‐6825‐5p	*hsa‐miR‐6785‐5p*	*hsa‐miR‐149‐3p*	*hsa‐miR‐5096*
CircRNA.16	*hsa_circ_0005741*	Chr10	Exon	0.00806	8.6005	Up	FBXW4	42	*hsa‐miR‐4254*	*hsa‐miR‐6837‐3p*	*hsa‐miR‐6813‐3p*	*hsa‐miR‐4518*	*hsa‐miR‐6768‐3p*
CircRNA.6559	*hsa_circ_0003928*	Chr17	Exon	0.00663	8.5685	Up	TAOK1	34	*hsa‐miR‐3692‐5p*	*hsa‐miR‐501‐5p*	*hsa‐miR‐7151‐5p*	*hsa‐miR‐516b‐5p*	*hsa‐miR‐6838‐3p*
CircRNA.1617	*hsa_circ_0003712*	Chr11	Exon	0.02142	7.5248	Up	IMMP1L	97	*hsa‐miR‐3139*	*hsa‐miR‐28‐5p*	*hsa‐miR‐4763‐5p*	*hsa‐miR‐6747‐3p*	*hsa‐miR‐1273g‐3p*
CircRNA.11877	*hsa_circ_0007312*	Chr22	Exon	0.04885	7.5076	Up	SPECC1L	18	*hsa‐miR‐4763‐3p*	*hsa‐miR‐1322*	*hsa‐miR‐3591‐3p*	*hsa‐miR‐1909‐3p*	*hsa‐miR‐7150*
CircRNA.16349	*hsa_circ_0070022*	Chr4	Exon	0.02773	7.4609	Up	SDAD1	22	*hsa‐miR‐4709‐3p*	*hsa‐miR‐514a‐5p*	*hsa‐miR‐6800‐3p*	*hsa‐miR‐302c‐5p*	*hsa‐miR‐526b‐5p*
CircRNA.7693	*hsa_circ_0006918*	Chr18	Exon	0.00894	7.4314	Up	MIB1	16	*hsa‐miR‐4652‐3p*	*hsa‐miR‐6838‐3p*	*hsa‐miR‐218‐1‐3p*	*hsa‐miR‐6847‐5p*	*hsa‐miR‐1273g‐3p*
CircRNA.13503	*hsa_circ_0005579*	Chr2	Exon	0.01308	7.3058	Up	CRIM1	44	*hsa‐miR‐383‐3p*	*hsa‐miR‐146‐5p*	*hsa‐miR‐3616‐3p*	*hsa‐miR‐3973*	*hsa‐miR‐488‐3p*

### RT‐qPCR verified the selected differentially expressed circRNAs in high glucose‐induced HUVECs

**Figure 3:**
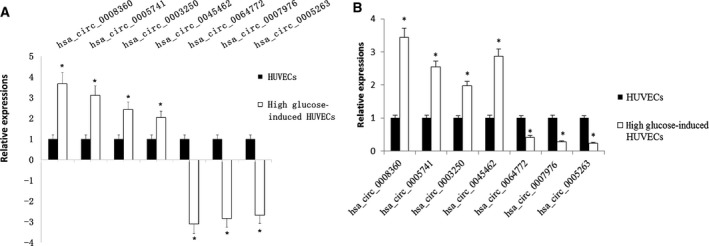
Verification of the differentially expressed circRNAs by RT‐qPCR. (A‐B) The relative expression levels of seven selected circRNAs in control HUVECs and high glucose‐induced HUVECs, as measured by RNA‐seq (A) and qRT‐PCR (B). The expression levels of all selected circRNAs were normalized to 1. *n* = 3 for each group; The statistical test is Student’s t‐test, and the error bars represent SD; *, *P* < 0.05, compared with the control group.

Next, we randomly selected seven differentially expressed circRNAs for RT‐qPCR verification. These candidates included four top 10 up‐regulated circRNAs and three top 10 down‐regulated circRNAs. As shown in Fig. 3A,B, the qPCR results confirmed the up‐regulation of *hsa_circ_0008360* (3.44‐fold in the qPCR, 12.71‐fold in the RNA‐seq), *hsa_circ_0005741* (2.54‐fold in the qPCR, 8.60‐fold in RNA‐seq), *hsa_circ_0003250* (1.98‐fold in the qPCR, 5.37‐fold in the RNA‐seq), and *hsa_circ_0045462* (2.87‐fold in the qPCR, 4.09‐fold in the RNA‐seq) and down‐regulation of *hsa_circ_0064772* (2.38‐fold in the qPCR, 8.59‐fold in the RNA‐seq), *hsa_circ_0007976* (3.57‐fold in the qPCR, 7.14‐fold in the RNA‐seq), and *hsa_circ_0005263* (4.35‐fold in the qPCR, 6.39‐fold in the RNA‐seq) in high glucose‐induced HUVECs. The consistency between the results of RNA‐seq and RT‐qPCR indicated the high reliability of the high‐throughput RNA‐seq approach for screening differentially expressed circRNAs in our study.

### Characterizations of the parental genes of differentially expressed circRNAs

Although circRNAs, unlike traditional linear mRNAs, are synthesized via backsplicing, both circRNAs and mRNAs are generated from mRNA precursors (pre‐mRNA). Thus, characterizing the function of corresponding linear mRNAs is the common way to enhance our understanding of the features of circRNAs. In this study, we identified 214 differentially expressed circRNAs, 20 of which were intergenic circRNAs with no parental genes. The remaining 194 circRNAs consisting of circular exonic RNAs and intron circRNAs were originated from 159 parental genes. All of these parental genes were further subjected to analyses with clusterProfilers, including Gene Ontology (GO) and Kyoto Encyclopedia of Genes and Genomes (KEGG) pathway enrichment.

**Figure 4:**
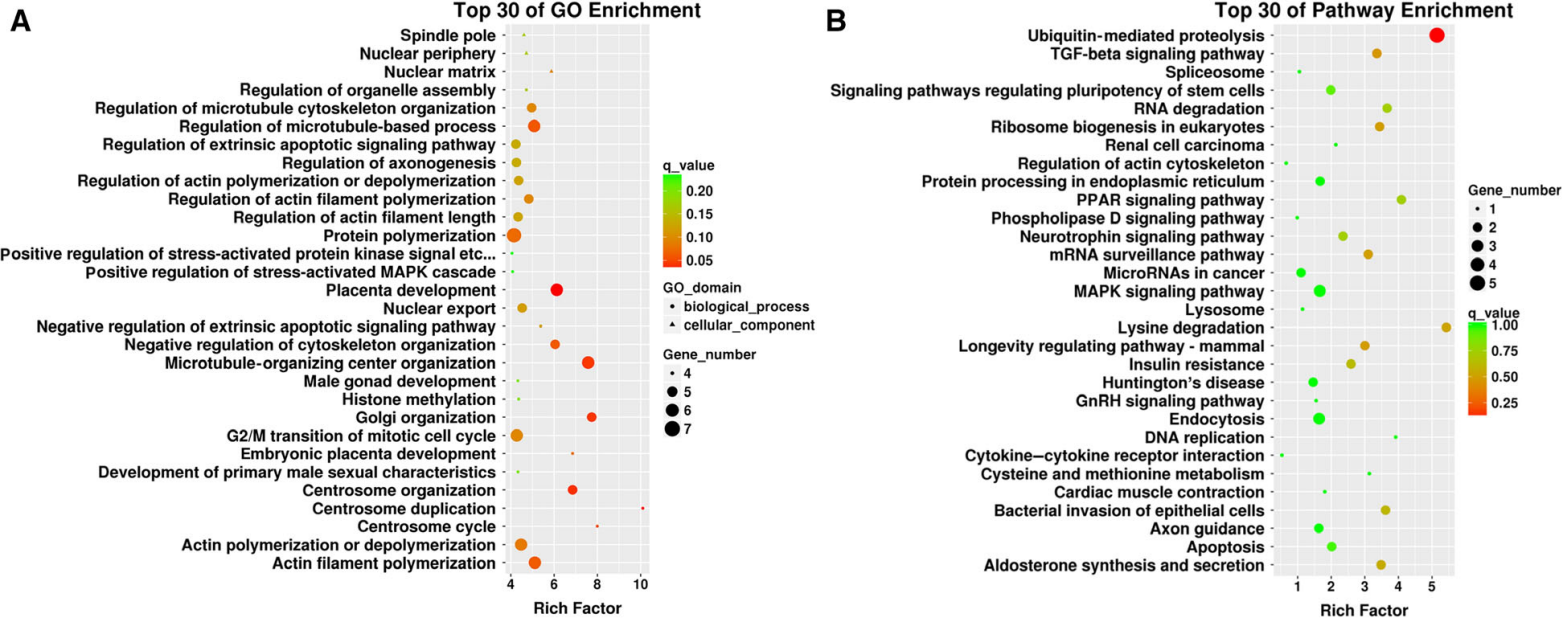
Functional profiles of the parental genes of differentially expressed circRNAs in high glucose‐induced HUVECs. (A, B) 159 parental genes from 194 differentially expressed circRNAs were subjected to GO and KEGG pathway enrichment analyses. The top 30 classes of GO enrichment terms (A) and top 30 classes of KEGG pathway enrichment terms (B) are presented. The gene numbers are indicated by the sizes of dots, while the *q*‐value is denoted by the color of dots.

The top five most enriched and meaningful biological process terms were related to ‘cytoskeleton organization’, ‘organelle organization’, ‘microtubule organizing center organization’, ‘mitotic cell cycle’, and ‘Golgi organization’. The top five most enriched cellular component terms were associated with ‘nuclear lumen’, ‘nucleus’, ‘microtubule cytoskeleton’, ‘microtubule organizing center’, and ‘spindle’. For the molecular function (MF) categories, we found that the most enriched MF terms were closely related to ‘ligase activity’, ‘ubiquitin‐protein transferase activity’, ‘ubiquitin‐like protein transferase activity’, ‘zinc ion binding’, and ‘poly(A) RNA binding’ (Fig. 4A). Moreover, the KEGG pathway analysis revealed the top 30 pathways that might function in high glucose‐induced HUVECs (Fig. 4B). The most enriched and meaningful pathways were related to ‘Ubiquitin mediated proteolysis’, ‘Lysine degradation’, ‘peroxisome proliferator‐activated receptor signaling pathway’, ‘RNA degradation’, and ‘Bacterial invasion of epithelial cells (hsa05100)’.

### Prediction of target miRNAs of the top differentially expressed circRNAs and construction of a representative circRNA–miRNA network

Recent studies on circRNA/miRNA interaction have indicated that circRNAs play a key role in the regulation of gene expression by interacting with their target miRNAs. To find the potential target miRNAs that may be associated with high glucose induction, we first predicted the complementary miRNAs that correlated with the top 10 up‐regulated and top 10 down‐regulated differentially expressed circRNAs using a miRanda‐based software (Table S1). The correlation degrees of the 20 differentially expressed circRNAs (Table S2) and 848 complementary miRNAs (Table S3) were also calculated.

**Figure 5:**
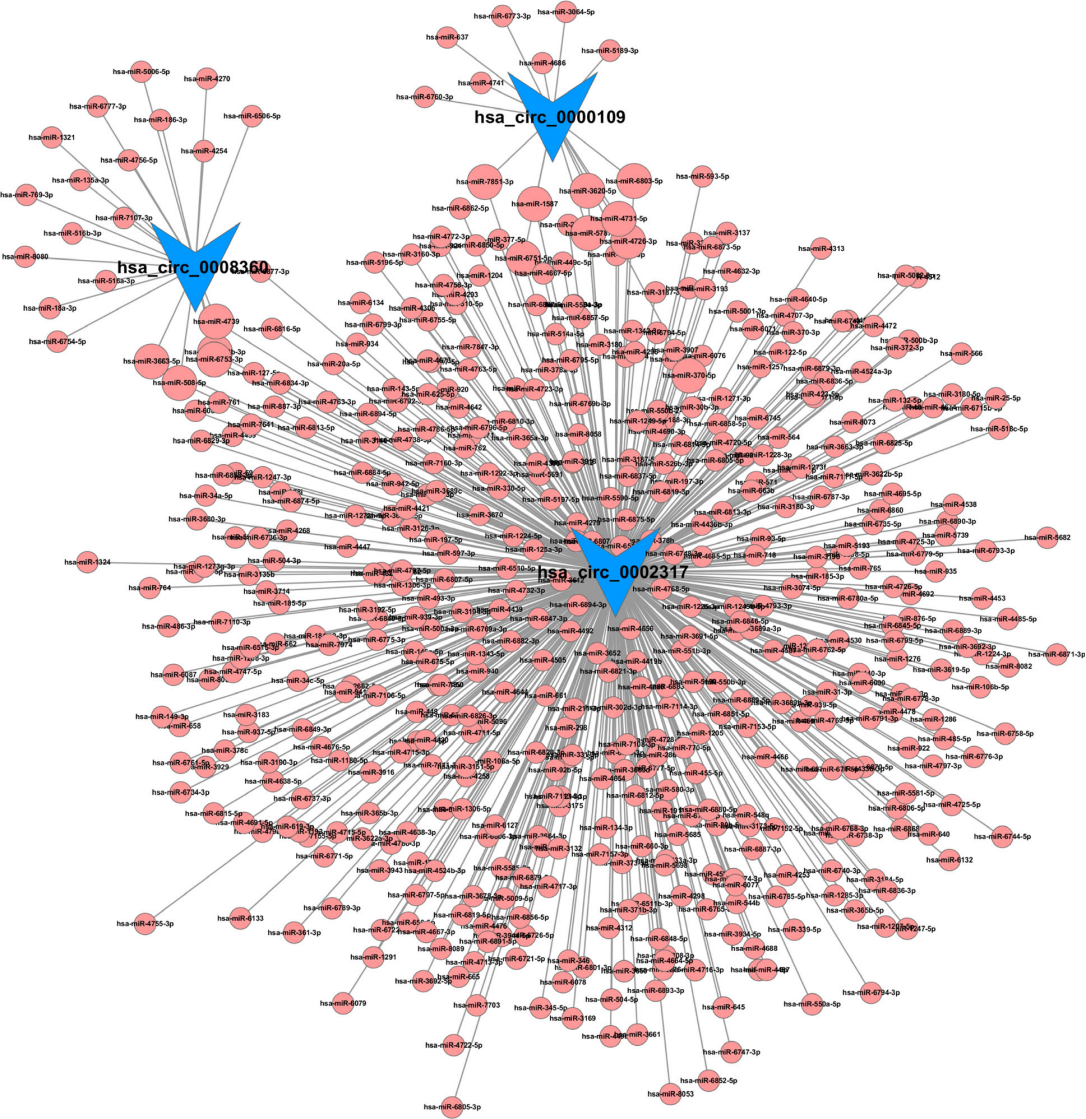
A representative circRNA–miRNA network constructed from three most differentially expressed circRNAs and their putative target miRNAs. Among all the differentially expressed circRNAs, *hsa_circ_0008360*, *hsa_circ_0000109,* and *hsa_circ_0002317* (indicated by vee nodes) had the highest magnitude of difference in high glucose‐induced HUVECs. They were predicted to be functionally connected with their targeted miRNAs (indicated by circles) in the network. Detailed correlations between circRNAs and putative miRNAs are shown in Table S1, while the degree distributions of circRNA nodes and miRNA nodes are presented in Table S2 and Table S3, respectively.

The top five putative MREs for the top 10 up‐regulated and down‐regulated known circRNAs are listed in Table 2, and *hsa_circ_0002317*, *hsa_circ_0087015,* and *hsa_circ_0056891* have more 100 MREs. Meanwhile, *hsa‐miR‐4656*, *hsa‐miR‐3620‐5p*, *hsa‐miR‐1587*, *hsa‐miR‐93‐3p*, and *hsa‐miR‐6877‐3p* can be targeted by five circRNAs. Among these differentially expressed circRNAs, *hsa_circ_0008360*, *hsa_circ_0000109,* and *hsa_circ_0002317* had the highest magnitude of difference in high glucose‐induced HUVECs, and they exhibited over ninefold of expression alteration. Therefore, these three circRNAs with most significantly differential expressions between high glucose‐induced and control HUVECs, as well as their putative target miRNAs, were used to construct a representative circRNA–miRNA network (Fig. 5).

## Discussion

Circular RNAs identified in humans, animals, plants (including Arabidopsis, rice, and soybean), and yeasts (*Saccharomyces cerevisiae* and *Schizosaccharomyces pombe*) 31, 32, 33, 34, 35 can serve as sponges of miRNAs and regulate the expression of parental genes 14, 22, 36, 37. The roles of circRNAs have been reported in various diseases including diabetes and many vascular diseases 38, 39. Particularly, some studies have identified the functions of certain circRNAs in vascular endothelial cells induced by hypoxia or other inducers 40, 41, 42. In this study, we performed a comprehensive profiling of differentially expressed circRNAs in high glucose‐induced HUVECs, and our subsequent integrated bioinformatic analyses revealed potential pathways correlated to the pathogenesis of endothelium dysfunction caused diabetes. For example, KEGG analysis of circRNAs correlated with parental genes indicated the activation of ubiquitin‐mediated proteolysis and transforming growth factor‐β signaling pathway in high glucose‐exposed HUVECs.

Compared with control HUVECs, high glucose‐induced HUVECs expressed 130 significantly up‐regulated (the top five were as follows: circRNA.12079, circRNA.9089, circRNA.5846, circRNA.16, and circRNA.6559) and 84 significantly down‐regulated circRNAs (the top five were as follows: circRNA.15166, circRNA.7966, circRNA.3075, circRNA.12564, and circRNA.4470). Seven differentially expressed circRNAs, which have been reported to be expressed in the human frontal cortex 18, were randomly selected for RT‐qPCR verification. The results of RT‐qPCR verification suggested the high reliability of the high‐throughput RNA‐seq in screening circRNA with significantly altered expressions. Because *hsa_circ_0008360*, *hsa_circ_0000109*, and *hsa_circ_0002317* had the highest magnitude of altered expressions in the study, we took them as candidates to analyze their functions and probe the potential mechanisms involved in endothelial dysfunction. Targeted miRNA prediction of these three circRNAs (Fig. 5) showed that they all have many target miRNAs, some of which have been reported to function in the biology of vascular endothelium in previous studies.

In our study, *hsa_circ_0008360* had been validated to be more expressed in high glucose‐induced HUVECs, and it was predicted as a sponge of *hsa‐miR‐186‐3p* (Table 2). The previous study showed that *miR‐186‐5p* was up‐regulated in patients with prostate cancer and inhibition of *miR‐186‐5p* could suppress cells proliferation, anchorage‐independent growth, and invasion of prostate cancer cells by increasing expression of tumor suppressor target gene A‐kinase anchor protein 12 43. Jiang *et al.* 44 demonstrated that the distribution of *miR‐186‐5p* contributed to high glucose‐induced cytotoxicity and apoptosis in AC16 cardiomyocytes. Recently, it has been confirmed that miR‐186 suppresses the proliferation and migration ability of HUVECs, while it enhances the apoptosis of HUVECs by targeting hypoxia‐inducible factor‐1α 41. However, whether *hsa_circ_0008360* regulates the proliferation and apoptosis of vascular endothelial cell by targeted regulation of *miR‐186* and the key mRNAs involved in these processes needs further investigation. In addition, other predicted target miRNAs of *has_circ_0008360* have also been reported to be regulators of genes related to proliferation or functions in vascular endothelial cells. For instance, Yuan *et al.* 45 suggested that *miR‐18a* regulated coronary heart disease development through targeting estrogen receptor. *MiR‐135a‐3p* has been revealed to function as a pivotal regulator of pathophysiological angiogenesis and tissue repair by targeting a vascular endothelial growth factor (VEGF)–p38K signaling axis 46. Therefore, *has_circ_0008360* may regulate the functions of endothelial cells by targeting a variety of miRNAs.

*Hsa_circ_0000109* was the second most up‐regulated circRNA in HUVECs exposed to high glucose stimulation. Among its putative target miRNAs, *miR‐370* is the one with close association with the proliferation, apoptosis, and angiogenesis of vascular endothelial cells. The proliferation of HUVECs and formation of capillary‐like structures can be promoted by *miR‐370* via inhibiting the expression of forkhead box 1 and maternally expressed gene 3 47, 48, 49. But in retinal capillary endothelial cell, *miR‐370* was reported to induce growth inhibition and apoptosis by reducing the expression of kinase insert domain‐containing receptor gene 50. Such discrepancies mechanism could be due to the contexts of different endothelial cells. Some other predicted target miRNAs of *hsa_circ_0000109*, including *miR‐637*, *miR‐3620‐5p*, *miR‐3064‐5p*, and *miR‐6803‐5p*, have not been reported directly related to functional regulations of HUVECs. But these miRNAs have been unveiled to play central roles in the competing endogenous RNA (ceRNA) network in others diseases 51, 52, 53, 54. However, they might be new targets in ceRNA network in vascular endothelium damage caused by high glucose.

*Hsa_circ_0002317* is another circRNA that was among the most up‐regulated circRNAs in HUVECs under high glucose condition. Bioinformatics prediction suggested that the target miRNAs of *hsa_circ_0002317* were very abundant. For examples, Ru *et al.* demonstrated that *miR‐149‐3p* was involved in the mechanisms of voltage‐gated K^+^ channels in mediating cell proliferation and apoptosis in human glioma U87‐MG cells 55, but the specific regulatory molecule mechanism was not clear. In tumor endothelial cells, *miR‐149‐3p* facilitated the activation of nuclear factor kappa B (NF‐κB) signaling and promoted expression of pro‐inflammatory and pro‐angiogenic factors 56. Chamorro‐Jorganes *et al.* reported that both *miR‐149* and miR‐149* up‐regulated angiogenic responses by regulating fibroblast growth factor 2 but lowered the number of HUVECs 57. Likewise, expression of *miR‐93‐5p* was confirmed to be correlated with the up‐regulations of proliferation and angiogenesis by targeting VEGF or cell cycle regulatory pathways 58, 59, 60. Therefore, the interaction between *hsa_circ_0002317* and its target miRNAs might contribute to the mechanisms that include multiple signaling pathways. However, due to different conditions of cell culture and treatment in these studies, these mechanisms cannot be fully applicable to our study. Precisely mapping the network with well‐characterized upstream and downstream miRNAs and mRNAs remains to be fulfilled to elucidate the molecular mechanisms underlying vascular endothelium repairment after high glucose insult. Conducting the investigation on the functions of *hsa_circ_0002317*, *miR‐93‐5p* and their interaction in the biology behavior of endothelial cells may help identify new therapeutic targets of diabetic vascular lesions.

## Conclusion

In summary, our work on circRNA profiling and target miRNAs predictions supports that differentially expressed circRNAs in high glucose‐induced HUVECs play important roles in mitigating functional and angiogenic damage. Some crucial circRNAs, such as *hsa_circ_0008360*, *hsa_circ_0000109,* and *hsa_circ_0002317*, can regulate the expressions of multiple genes involved in vascular endothelial function and angiogenesis through targeting miRNAs. These findings help us better understand the underlying mechanisms of circRNAs in preventing high glucose‐induced dysfunction of vascular endothelial cells. Further study of the functions of these circRNAs can illuminate the profound mechanisms of endothelial dysfunction induced by hyperglycemia and thus sheds new light on developing new interventions for diabetic vascular complications.

## Conflict of interest

The authors declare no conflict of interest.

## Author contributions

GXJ and MLL designed the study; XLL and XLH performed the experiments; EQX and QW carried out the data analysis; GXJ and XYP wrote the manuscript; and all authors read and approved the final manuscript.

## Supporting information


**Table S1.** The list of predicted complementary miRNAs that correlated with the top 10 up‐regulated and top 10 down‐regulated differentially expressed circRNAs in high glucose‐induced HUVECs.

**Table S2.** The list of correlation degrees of top 10 up‐regulated and top 10 down‐regulated differentially expressed circRNAs.

**Table S3.** The list of correlation degrees of 848 putative complementary miRNAs.

## Data Availability

The datasets used in this study are available from the corresponding author on reasonable request.
